# Deafness, Its Causes, Prevention and Cure

**Published:** 1829-01-01

**Authors:** 


### DEAFNESS; ITS CAUSES, PREVENTION AND CURE. By *John Stevenson*, Esq. 8vo.

No one will dispute the importance of the organ of hearing to the welfare as well as the pleasure of man—and as the disorders of this organ are very common, and all ranks of society are now dabbling in physic and surgery, we do not much wonder that Mr. Stevenson should have given his Treatise a *popular* form. There was no occasion, however, to tell his readers so in the introduction. The general reader prefers hard words, because he does *not* understand them—the professional reader prefers them because he does; and the *latter* is usually prejudiced against all ostensible attempts to enlighten the non-professional public on medical matters. That popular medical works do harm, there can be little doubt—but the question is, do they not, also, some good? We believe they do. If the non-professional public did not acquire a smattering of medical science, many practitioners would have a temptation to rest satisfied with a smattering also. They are now forced to keep their distance before the general reader, in order to avoid unpleasant collisions, and maintain their superiority.

But a medical writer, when he expressly tells us that he addresses himself to the general as well as to the professional public, has no claim on any extended notice of his work in a purely medical review. This Mr. Stevenson must bear in mind.

We shall pass over the first half of the volume, which treats of the anatomy and physiology of the ear, addressed (and improperly addressed) to the general reader. In the fourth chapter Mr. S. takes up the DISEASES of the organ; but here again we are met by a *damper*.

"Persuaded, as I am, that if 'a little learning is a dangerous thing' in reference to subjects of literature, it is tenfold more so when applied to the healing art, I shall studiously avoid the error into which writers on popular medicine are too frequently betrayed; namely, that of detailing histories which cannot be understood, and of prescribing formulæ for powerful remedies which cannot be used with impunity by unprofessional readers. Instead of adopting a plan calculated to mislead or injure rather than benefit those who seek to be instructed, by arming them with a two-edged sword, which they cannot wield without hazard of wounding themselves, I propose to detail the symptoms which indicate danger, or require for their treatment the hand of experience;—to point out the various and often unsuspected causes of local derangement;—to show how far the patient may, without risk, be guided by his own acquired knowledge;—and lastly, to caution him against the employment of useless or dangerous domestic nostrums, suggested by persons alike ignorant of the nature and character of disease, and of the action and effects of those *remedies* which they so fearlessly recommend." 117.

It is curious that wounds, tumours, and ulcers, which partially destroy the texture, do not materially impair the function of the AURICLE. Even its entire removal only occasions a temporary confusion of hearing. The most troublesome and distressing ailment to which the auricle is liable, is an herpetic eruption, consisting of numerous small watery pimples, or vesicles, surrounded by an inflamed base.

"These little vesicles bursting spontaneously, or being more frequently ruptured by the fingers of the patient—who is almost irresistibly impelled to rub or scratch them, with a view to allay the accompanying almost intolerable smarting and itching—they pour out a copious discharge, which soon becoming fœtid and acrimonious, occasions irritation, excoriation, and often ulceration of the affected surface.

"If the progress of this disease be not speedily arrested, the skin and subjacent cellular texture begin to thicken and enlarge to such a degree, as to render the auricle, already inflamed, disgustingly frightful and deformed. Nor is this the termination of the mischief.—In consequence of the tumefaction extending to the soft parts of the auditory canal, and of the inspissation of the discharge, the area of this tube becomes so much narrowed, and in some instances so completely obliterated, as to offer a considerable barrier or total obstruction to the ingress of sound, causing, while the disorder continues, either partial or total deafness." 126.

What will the professional reader think when he is told that the adaptation of local and general remedies is much too difficult and complicated "*to be confided to any but the most experienced persons*"—and consequently it is kept back from the *profession*, as well as from the public! ! Can Mr. Stevenson justly complain, if we condemn this mode of procedure?—the very mode which DOCTOR, *alias* SURGEON HARRISON has adopted in his work on Crooked Spines.*

As a compensation for surgical information respecting HERPES AURICULÆ, Mr. Stevenson favours us with an anathema against hair-dressers, who "strip us of the pendent SIDE-LOCKS—the real ornaments and guardians of the ear"—also against flannel night-caps, evening parties in hot rooms, long dances, and exposure to currents of cold air, &c. We hope the ladies will lend an *ear* to these admonitions.

Speaking of otitis externa, Mr. S. observes, that frequent repetitions of the inflammation tend to render the membrane reflected over the tympanum, thick, dry, and opaque, as may be distinctly seen in a strong light:—

---

* The Doctor has addressed a long letter to Dr. Johnson in the LANCET, complaining of the review of his book of Demi-Quackery. The complainant also rips up an old grievance about a picture in the Exhibition at Somerset House; but he states no particulars. We shall help him on this occasion. The Doctor got his picture perched up in the exhibition, with a long *crooked spine* on one side, and his *intended* big book of CHARLATANNERIE, on the other! This we satirised as an advertisement—and the *crooked spine* has proved to be sufficiently emblematical of the Doctor's subsequent *crooked policy*.—ED.

"In this state of the affected part, a sense of cracking (crepitus) is sometimes experienced, accompanied with defective hearing—symptoms generally, but I suspect improperly, attributed to a want of wax, a privation which should be considered as operating *secondarily*, and injurious only by depriving the passage of its protecting secretion." 146.

Mr. S. properly condemns the application of stimulating and irritating substances in these cases—and enjoins the strictly antiphlogistic treatment.

In the reduction of otitis, Mr. S. recommends "cupping behind the auricle, or the local application of from two to six leeches behind, or on the concave surface of the ear*—fumigating the passage with the vapour of poppy-head tea, with a little hot vinegar, and afterwards applying to the opening of the meatus a dossil of lint imbued with warm sallad oil." Purgation is necessary, and the depletion should be repeated *pro re nata.* But otitis will sometimes baffle the most rigid antiphlogistics, and suppuration ensues. Fomentations, poultices, and generous diet, are then necessary. In these cases, spongy excrescences not unfrequently protrude into the meatus, and by plugging up the passage, keep up much irritation, in consequence of the retained matter.

Polypi grow on the lining membrane of the ear, in the same way as on the Schneiderian membrane. They may be extracted by the forceps—removed by scissors or ligature—and the part from which they are torn, touched with the nitrate of silver.

An adventitious membranous septum sometimes is found, external to the membrana tympani, and closing up the foramen. It may be congenital—or it may occur after long continued otorrhœa. It is ascertained by inspection in a strong light. Mr. S. met with a case where it was situated about a quarter of an inch outward from the membrana tympani. He punctured it, and hearing was immediately restored. This is the only method of cure.

### Redundancy or Deficiency of Cerumen.

Numerous causes concur to derange the secretion of the cerumen—among which Mr. S. enumerates, as a very frequent agent, "the use of *ear-pickers*, made of gold, silver, ivory, or some other hard substance." Sir Hans Sloane

---

* "In the case of the son of an eminent physician, a leech having been applied in front of the auricle for acute external inflammation of the ear, the temporal artery was penetrated, and required to be divided and tied, to suppress the dangerous hemorrhage that ensued." 147.

It is curious that neither Sir Astley Cooper, nor Mr. Stevenson, has stated this case correctly. Sir A. in his Lectures, says, the hæmorrhage was suppressed by pressure, though he himself was obliged to cut down and *divide* the artery, after which, pressure was sufficient. Mr. Stevenson is in error respecting *ligature* of the vessel. The accident, however, should render a surgeon cautious in the application of leeches in the direction of the temporal artery before the ear. The young gentleman alluded to, would have been destroyed in a few minutes more, had not Sir Astley cut down upon the artery.

published a paper on this subject, tracing almost all aural diseases to ear-picking.

"The symptoms characteristic of a diminution of hearing, proceeding from an imperfect or deficient secretion of wax, are—a dry, rustling sound, like the crackling of a distended bladder when handled, particularly during mastication; and an occasional ringing or depraved noise, with a dull sensation, in the ear. The sense of hearing is considerably deteriorated when the health and spirits are depressed, and in dull, moist, or cloudy weather—a change from which to a dry, clear, atmosphere restores the function of the organ to its former state of acuteness, by more powerfully conveying the undulations of sound to the membrane of the drum.

"With these occasional vicissitudes, the disease, though slowly so, is progressive, until at length the patient becomes sensible of a permanent diminution of hearing, amounting, in an unfavourable state of the air, to absolute deafness." 168.

These symptoms Mr. Stevenson refers to an arid state of the membrana tympani, rather than to the atonic condition of the ceruminous glands—and this he infers from the shining appearance exhibited by the septum, resembling dried parchment. The cause of the complaint has always been traced to the application of cold air, or to bathing at a time when the heat of the body had been raised to a high temperature. A chronic inflammation is set up, with interruption of the ceruminous secretion, and a gradual thickening of the investing membrane. The treatment consists in attention to the general health—defence against cold—

"And in instituting a *new and healthy action* in the secretory apparatus by a degree of warmth, *and medicated local remedies, adapted to the sensibility of the part affected.*" 170.

This, then, is the *treatment* which Mr. Stevenson has set before the general *and* the professional reader!

We should have supposed that the *crooked* policy of Doctor Harrison, which we so fully exposed, would have deterred all medical writers, in future, from following such a bad example. We are sorry to see a man like Mr. Stevenson, fall into such an impolitic mode of book-making. We respect his talents, and we honour his private character; but as public Journalists, we must and will express our disapprobation of the course here pursued.

If Mr. Stevenson had been a young adventurer, or a bankrupt practitioner, we might have pitied the necessity which dictated the recourse to such a measure, and dropped the pen. But Mr. Stevenson is in the full career of a lucrative practice, and therefore, deserves the rigour of criticism for descending to the miserable shift of holding out that in the title-page which is not to be found in the body of the work.

Long experience has convinced us that our most rigid censors, whether well-wishers or enemies, are, in the long run, our best friends. Flattery is the deadliest of poisons—just criticism is the most salutary medicine for the mind. We know not in which class (well-wishers or enemies) Mr. Stevenson may place us; but if a life of *nearly half a century* has taught us any one truth more positive than all others, it is this—*that the test of friendship is the denunciation of our errors.*

